# Cutibacterium acnes Induces the Expression of Immunosuppressive Genes in Macrophages and is Associated with an Increase of Regulatory T-Cells in Prostate Cancer

**DOI:** 10.1128/spectrum.01497-21

**Published:** 2021-12-22

**Authors:** Sabina Davidsson, Jessica Carlsson, Larry Greenberg, Jonny Wijkander, Bo Söderquist, Ann Erlandsson

**Affiliations:** a Department of Urology, Faculty of Medicine and Health, Örebro University, Örebro, Sweden; b Department of Environmental and Life Sciences/Biology, Faculty of Health, Science and Technology, Karlstad University, Karlstad, Sweden; c Department of Health Sciences, Faculty of Health, Science and Technology, Karlstad University, Karlstad, Sweden; d School of Medical Sciences, Faculty of Medicine and Health, Örebro University, Örebro, Sweden; e Department of Laboratory Medicine, Faculty of Medicine and Health, Örebro University, Örebro, Sweden; Lerner Research Institute

**Keywords:** *Cutibacterium acnes*, macrophages, prostate cancer, regulatory T-cells, cancer, prostate

## Abstract

Tumors and infectious agents both benefit from an immunosuppressive environment. Cutibacterium acnes (*C. acnes*) is a bacterium in the normal skin microbiota, which has the ability to survive intracellularly in macrophages and is significantly more common in prostate cancer tissue compared with normal prostate tissue. This study investigated if prostate cancer tissue culture positive for *C. acnes* has a higher infiltration of regulatory T-cells (Tregs) and if macrophages stimulated with *C. acnes* induced the expression of immunosuppressive genes that could be linked to an increase of Tregs in prostate cancer. Real-time PCR and enzyme-linked immunosorbent spot assay (ELISA) were used to examine the expression of immunosuppressive genes in human macrophages stimulated *in vitro* with *C. acnes*, and associations between the presence of *C. acnes* and infiltration of Tregs were investigated by statistically analyzing data generated in two previous studies. The *in vitro* results demonstrated that macrophages stimulated with *C. acnes* significantly increased their expression of PD-L1, CCL17, and CCL18 mRNA and protein (*p <*0.05). In the cohort, Tregs in tumor stroma and tumor epithelia were positively associated with the presence of *C. acnes* (*P = *0.0004 and *P = *0.046, respectively). Since the macrophages stimulated with *C. acnes in vitro* increased the expression of immunosuppressive genes, and prostate cancer patients with prostatic *C. acnes* infection had higher infiltration of Tregs than their noninfected counterparts, we suggest that *C. acnes* may contribute to an immunosuppressive tumor environment that is vital for prostate cancer progression.

**IMPORTANCE** In an immune suppressive tumor microenvironment constituted by immunosuppressive cells and immunosuppressive mediators, tumors may improve their ability to give rise to a clinically relevant cancer. In the present study, we found that *C. acnes* might contribute to an immunosuppressive environment by recruiting Tregs and by increasing the expression of immunosuppressive mediators such as PD-L1, CCL17, and CCL18. We believe that our data add support to the hypothesis of a contributing role of *C. acnes* in prostate cancer development. If established that *C. acnes* stimulates prostate cancer progression it may open up avenues for targeted prostate cancer treatment.

## INTRODUCTION

Immune escape, defined by the incapacity of the immune system to eliminate tumor cells and thereby prevent clinically relevant cancer, is one of the hallmarks of cancer (1, 2). To avoid being eliminated, tumors can create an environment comprised of immune cells and immune-related proteins with immunosuppressive functions. The supportive mechanisms for an immunosuppressive environment are multifactorial, but one potentially strongly contributing factor is related to infectious agents that benefit from a dampened immune response in a similar manner to tumors.

Cutibacterium acnes (*C. acnes*) is a bacterium that can survive intracellularly in macrophages, enabling it to evade the host immune response (3). Several factors contribute to this intracellular persistence, such as catalase production and a thick tightly cross-linked cell wall (4). However, *C. acnes* also produces arginine deiminase (5), which can reduce host T-cell function (6). In line with this, it has been demonstrated that monocytes treated with interleukin (IL)-4, granulocyte-macrophage colony-stimulating factor (GM-CSF), and *C. acnes* induce T-cell differentiation to immunosuppressive regulatory T-cells (Tregs) (7). *C. acnes* can induce the expression of both pro- and anti-inflammatory cytokines (8), but despite the possible link between *C. acnes* and increase of Tregs, few studies have focused on the anti-inflammatory effects of *C. acnes.* However, it has been shown that *C. acnes* induces IL-10 expression in adherent peripheral blood mononuclear cells; that is, monocytes (9).

*C. acnes* normally constitutes part of the microbiota on human skin and mucous membranes but can cause insidious long-lasting chronic infections at various sites including the prostate (3, 10–12). It has also been identified as the predominant microorganism in prostatic tissue specimens obtained from prostate cancer patients (8, 13, 14). *C. acnes* has been classified into different subtypes (IA, IB, IC, II, and III), and genotyping has revealed that all subtypes are capable of colonizing the prostate although type II has been reported as the most prevalent subtype (8, 11, 14).

The most abundant immune cell in the tumor environment is the macrophage, which has the ability to polarize into a tumor-inhibiting M1 phenotype or an M2 phenotype with tumor-promoting and immunosuppressive functions (15, 16). A number of different immuno-mediators, such as programmed death-ligand 1 (PD-L1), macrophage colony stimulation factor (M-CSF), C-C motif chemokine ligand 2 (CCL2), C-C motif chemokine ligand 17 (CCL17), C-C motif chemokine ligand 18 (CCL18) and C-C motif chemokine ligand 22 (CCL22), predominantly released by M2 macrophages, have been linked to increased numbers of both macrophages and Tregs in tumor tissue (15, 17–19). An immune-suppressive tumor environment constituted by Tregs and M2 macrophages along with immunosuppressive mediators such as CCL17, CCL18, CCL22, and PD-L1 can assist tumor development and progression (20, 21). In prostate cancer, higher infiltration of Tregs and M2 macrophages in tumor tissue has been associated with worse prognosis (1, 2, 22–25).

The aim of the present study was to investigate if higher levels of Tregs in prostate cancer tissue is associated with the presence of *C. acnes* and whether macrophages stimulated with *C. acnes* increase their expression of immunosuppressive genes that contribute to recruitment of Tregs in prostate cancer.

## RESULTS

### *C. acnes* stimulated macrophages.

**FIG 1:**
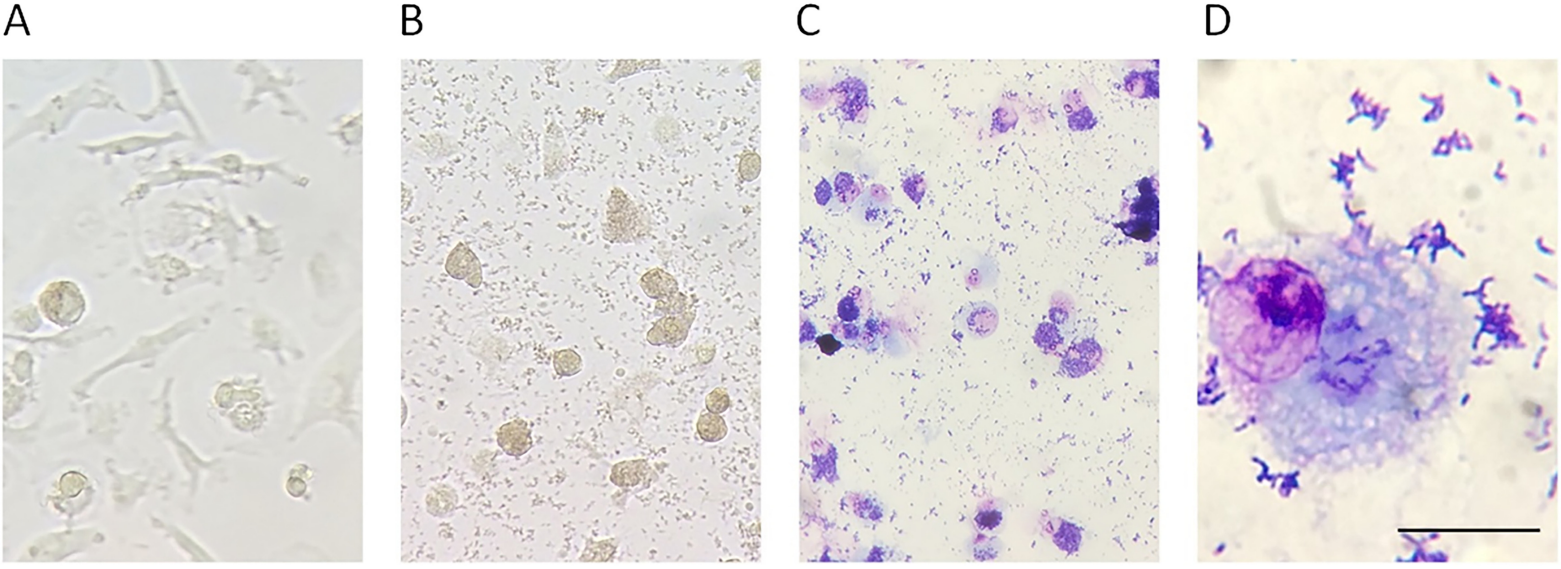
Representative images of the change of macrophage morphology after stimulation with *C. acnes* type II. (A) Unstimulated macrophages in culture plate after 48 h of incubation (x20). (B) Macrophages in culture plate stimulated with 10 MOI *C. acnes* type II for 48 h of culture (x20). (C) Macrophages stimulated with 10 MOI *C. acnes* type II for 48 h of culture followed by Giemsa staining (x20). (D) Macrophages stimulated with 10 MOI *C. acnes* type II for 48 h of culture, scraped off the culture plate and stained with Giemsa staining (×100, scale bar 30 μm).

To investigate the impact of *C. acnes* on secretion of immunosuppressive mediators from macrophages, we stimulated macrophages obtained from healthy male donors with *C. acnes* strains isolated from prostate cancer tissue. We selected a macrophage and *C. acnes* ratio of 10 multiplicity of infection (MOI) based on previous experiment incubating *C. acnes* with cultured prostate cancer cells (14). After a 48-h incubation of M0 macrophages with 10 MOI *C. acnes* of type IA or II, the macrophages had transformed from an elongated shape to a rounder shape with a foamy appearance. At this point, *C. acnes* of both type IA or II, had increased in number (Fig. 1A–D).

### mRNA and protein expression in macrophages stimulated with *C. acnes*.

**FIG 2:**
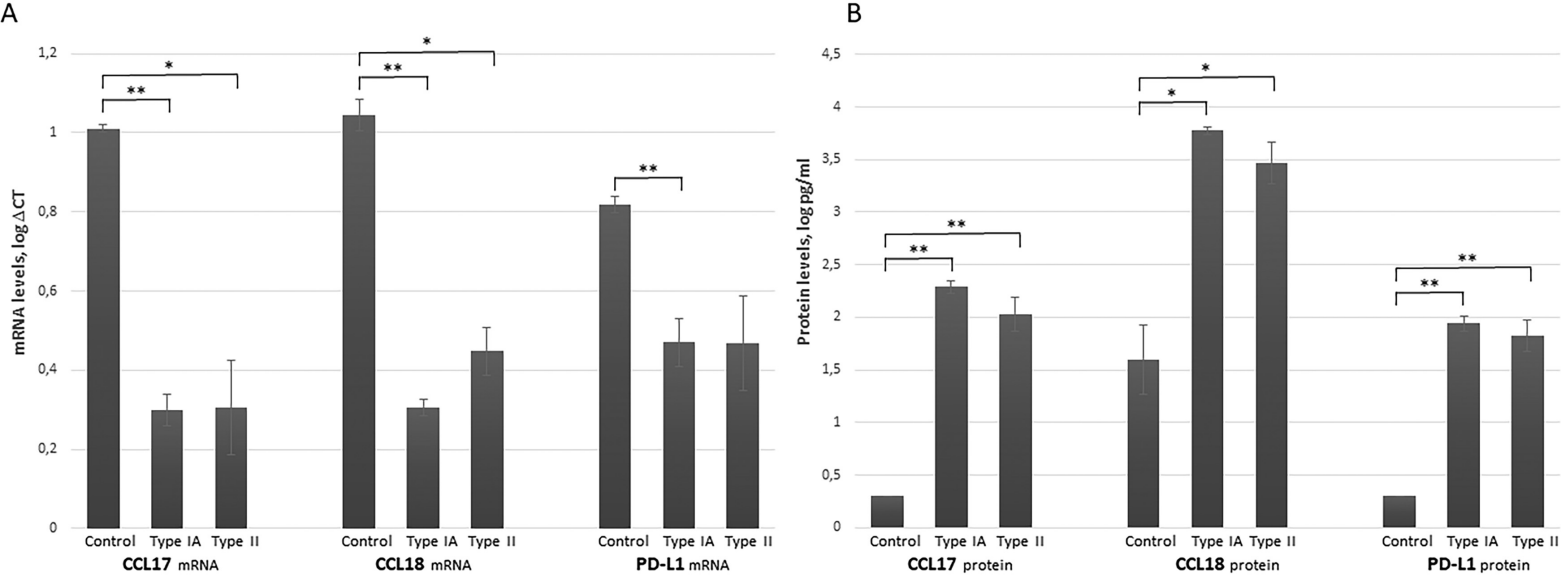
Expression of CCL17, CCL18, and PD-L1 in unstimulated macrophages (control) and macrophages stimulated with *C. acnes* type IA or type II. (A) mRNA expression in unstimulated control and *C. acnes* type IA or type II stimulated macrophages, presented as mean +/- SD of log ΔCT (i.e., the mRNA expression of the gene of interest minus the expression of the housekeeping gene POLR2F). The lower the ΔCt value, the higher the expression of the mRNA of interest. (B) Protein expression in unstimulated control and *C. acnes* type IA or type II stimulated macrophages, presented as mean +/- SD of log pg/ml. The statistical analysis were performed using repeated measurements ANOVA with two within subject factors (treatment and replicates). The treatments were unstimulated (control), stimulated with *C. acnes* type IA or with *C. acnes* type II. The number of replicates were three, done with three different macrophage donors. *P < 0.05, **P < 0.01.

Stimulation of macrophages with *C. acnes* type IA or type II produced a significantly increased mRNA expression of CCL17 (type IA: *P = *0.006; type II: *P = *0.029) and CCL18 (type IA: *P = *0.002, type II: *P = *0.023). Stimulation with type IA also produced a significant increase in PD-L1 (*P = *0.032). The effects of the stimulation on the mRNA expression level are presented in Fig. 2A as log ΔCt values. The fold change increases in the mRNA expression in the stimulated macrophages compared with their unstimulated counterparts were 100–660 for CCL17, 190–790 for CCL18, and 2–40 for PD-L1. No significant differences were seen in expression of the mRNA encoding M-CSF, CCL2, and CCL22 (*P > *0.05) (Supplement 1 in the supplemental material). There were also no significant difference in expression of any of the mRNA between the macrophages stimulated with *C. acnes* type IA and those stimulated with type II.

For the genes that demonstrated a significant increased expression of mRNA (i.e., *PD-L1*, *CCL17*, and *CCL18*), enzyme-linked immunosorbent spot assays (ELISAs) were performed to confirm significant changes in protein levels in the culture media. Macrophages stimulated with *C. acnes* type IA or type II demonstrated increased levels of all three proteins: CCL17 between 97–289 pg/ml (type IA: *P = *0.001; type II: *P = *0.009), CCL18 between 560–9148 pg/ml (type IA: *P = *0.022; type II: *P = *0.041), and PD-L1 between 28–147 pg/ml (type IA: *P = *0.002; type II: *P = *0.009) proteins (Fig. 2B). There were no significant differences in protein levels between the macrophages stimulated with *C. acnes* type IA and those stimulated with type II (*P > *0.05).

### Associations between infiltration of Tregs and *C. acnes* in prostate tissue from patients with prostate cancer.

Associations between the presence of *C. acnes* and infiltration of Tregs were investigated by statistically analyzing data generated in two previous studies investigating the presence of *C. acnes* (14) and Tregs (24), respectively, in the same cohort of patients. Prostate cancer is generally multifocal with tumors presenting histological differences. In the present study, we analyzed the association between the presence of *C. acnes* and Tregs in 151 tumors of the prostate gland obtained from 137 prostate cancer patients. Among these 137 men, 73 cases were *C. acnes* positive and 64 cases were *C. acnes* negative, the 14 additional tumors from these 137 men were from 10 *C. acnes* negative and 4 *C. acnes* positive cases with multiple tumors. The new statistical analysis performed in the present study demonstrated that Tregs, both in the tumor stromal and the tumor epithelial compartment, were positively associated with the presence of *C. acnes* (*P = *0.0004 and *P = *0.046, respectively) (Table 1).

**TABLE 1:** Infiltration of Tregs in 151 tumors (stromal and epithelial compartment) from 137 prostate cancer patients with and without prostatic *C. acnes* infection

Infiltration of Tregs	Patients with no *C. acnes*	Patients with *C. acnes*
Tregs in tumor stroma (%)		
Yes	17 (30)	39 (70)
No	57 (60)	38 (40)
		
Tregs in tumor epithelia (%)		
Yes	15 (33)	30 (67)
No	59 (56)	47 (44)

## DISCUSSION

An increasing number of studies have demonstrated that chronic infections are often associated with increased infiltration of anti-inflammatory cells, and that this infiltration can support cancer initiation and progression (8, 26, 27). In the present study, we investigated whether *C. acnes* was able to induce the expression of immunosuppressive proteins in macrophages, which are among the most abundant immune cells in tumor environments. Our results showed that macrophages cultured with *C. acnes* increased their expression of three proteins that are involved in recruitment and accumulation of Tregs *in vivo*. In addition, we found that the number of Tregs was significantly higher in prostate cancer tissue that was culture-positive for *C. acnes* compared with nonculture-positive tissue.

M2 macrophages and Tregs are immunosuppressive cells that under normal conditions prevent autoimmune reactions and excessive inflammation. The side effect of this immunosuppression is the loss of both anti-tumor immunity and effective elimination of chronic bacterial, viral, or fungal infections (15, 28–30). In the present study, we investigated the expression of mRNA encoding M-CSF, CCL2, CCL17, CCL18, CCL22, and PD-L1 in macrophages stimulated with *C. acnes*. The main function of M-CSF, CCL2, and CCL22 is to recruit M2 macrophages and/or Tregs. In our study, the expression of these genes in the macrophages was already high at a basal level, and no significant increase could be determined (Supplement 1). This may have been due to the pretreatment of monocytes with M-CSF that we performed *in vitro* for the differentiation of monocytes into macrophages. However, a significantly increased expression of both mRNA and the corresponding proteins regarding CCL17, CCL18, and PD-L1 was observed in macrophages stimulated with *C. acnes*. These genes, *CCL17*, *CCL18*, and *PD-L1*, are mainly expressed in M2 macrophages, indicating that *C. acnes* is able to polarize the macrophage into an immunosuppressive M2 phenotype with the ability to recruit Tregs (15). In line with our results, a recently published study on gastric cancer identified *C. acnes* as a possible cause of increased polarization of macrophages against an M2 phenotype and increased cancer progression (31). Our microscopic examination of the macrophages stimulated with *C. acnes* revealed a rounder shape with a foamy appearance, as previously demonstrated in M2 macrophages with PD-L1 expression (32). We did not detect any significant differences in expression of the genes between macrophages stimulated with *C. acnes* type IA and those stimulated with type II. However, other studies have found differences in the ability of *C. acnes* types to stimulate expression of immune-related genes (9). One study demonstrated a higher IL-10 expression in monocytes after stimulation with *C. acnes* type II compared with type I, though the induction of an interferon-γ response appeared to be similar between types I and II (9).

*C. acnes* is a Gram-positive, facultative anaerobic bacterium that constitutes part of the normal microbiota on human skin and mucosal membranes. Nevertheless, this opportunistic bacterium can cause both acute and low-grade chronic infections of greater or lesser severity (10–12). The main reason why *C. acnes* is able to evade the host immune response is its capacity to survive and proliferate intracellularly in macrophages, and it is worth noting that *C. acnes* has been suggested as an etiology of the granulomatous disorder seen in sarcoidosis (3, 12, 13, 33). One factor that can contribute to the intracellular survival of *C. acnes* is the production of catalase (4). In addition to catalase production, *C. acnes* has a cell wall with high resistance to degrading and oxidizing enzymes; and it also produces arginine dihydrolase (5), which contributes to depletion of arginine and subsequent reduced host T-cell function and proliferation as well as reduced development of an immunological memory (6). In line with this, it has been demonstrated that monocytes treated with IL-4, GM-CSF, and *C. acnes* induce a differentiation of T-cells into anti-inflammatory Tregs (7). However, it has been shown that *C. acnes* is able to induce the expression of both pro- and anti- inflammatory cytokines, with the pro-inflammatory reactions as a possible cause of DNA damage and initiation of tumorigenesis (8). IL-10 is an immunosuppressive interleukin that among other functions can stimulate differentiation of T-cells into Tregs (34). As already mentioned, it has been demonstrated that *C. acnes* can induce IL-10 expression in peripheral blood mononuclear cells, providing an opportunity for immune escape (9).

Mycobacterium tuberculosis (M. tuberculosis) is another example of a bacteria able to proliferate within macrophages. The main histological finding in tuberculosis, as for sarcoidosis, is granuloma with macrophages as the main cell type. In line with our results, macrophages stimulated with M. tuberculosis have increased PD-L1 and CCL18 expression (32, 35). Overexpression of PD-L1 correlates with worse prognosis in many cancers, including prostate cancer, and in some cancers there is an increasing use of therapeutic interventions that, for example, block PD-1 with pembrolizumab or PD-L1 with atezolizumab in order to reduce immunosuppression in the tumor environment (36).

High expression of CCL17 has been linked to worse prognosis in prostate cancer, renal cancer, and testicular cancer (18, 37, 38), and increased expression has also been observed in chronic viral infections such as hepatitis C and human papillomavirus, which are associated with malignant transformation (39, 40). High levels of CCL18 can be linked to cancer progression in colon cancer, breast cancer, and prostate cancer, where CCL18 not only activates recruitment of Tregs but also stimulates cancer cell migration and angiogenesis (17, 19, 41, 42). Increased expression has also been seen in gastric biopsies chronically infected with Helicobacter pylori and in nasopharyngeal carcinoma tissue with a latent infection of Epstein-Barr virus (43, 44).

The purpose of this study was to investigate whether *C. acnes* can induce the expression of immunosuppressive genes in macrophages, and whether there is an association between *C. acnes* and Tregs in prostate cancer. The study does have some important limitations. Only nine *in vitro* experiments were performed, using macrophages from three different blood donors, and the gene expression was examined using one specific ratio of 1:10 (10 MOI) of macrophage and live *C. acnes* at one time point. The most significant strengths are that the *in vitro* experiments were performed with *C. acnes* strains isolated from prostate cancer tissue and macrophages isolated from male blood donors, and the relatively large number of men included in the investigation of associations between Tregs and *C. acnes* infiltration.

This study found that macrophages stimulated with *C. acnes* increased the expression of genes involved in recruiting immunosuppressive Tregs. It also demonstrated significantly higher levels of Tregs in prostate cancer tissue with growth of *C. acnes* compared with culture-negative tissue. We suggest that *C. acnes* may contribute to an immunosuppressive status that facilitates a tumor-stimulating environment, which is vital for prostate cancer progression, and highlight the importance of further investigation regarding the role of infectious agents and other immune modifying factors in prostate cancer development and progression. In future studies, it is of great interest to investigate presence of *C. acnes* and expression of immunosuppressive proteins in normal prostate tissue and evaluate differences between men who develops prostate cancer compared with those without a prostate cancer diagnosis.

## MATERIALS AND METHODS

### Isolation of human monocytes and differentiation to M0 macrophages.

Buffy coats from healthy anonymous male blood donors were obtained from the Division of Clinical Immunology and Transfusion Medicine, Uppsala University Hospital, Uppsala, Sweden. Monocytes were isolated from the buffy coats by gradient centrifugation using Ficoll-Paque PLUS (GE Healthcare, Little Chalfont, UK). In short, about 50 ml of buffy coat was diluted with an equal volume of phosphate-buffered saline pH 7.4 containing 3 mM Ethylene diamine tetra acetic acid (PBS/EDTA), loaded on Ficoll-Paque PLUS, and centrifuged at 900 g for 30 min at 20°C. The separated mononuclear fraction was collected and diluted with PBS/EDTA followed by centrifugation at 500 g for 10 min. Cells were suspended in PBS/EDTA and washed four times with PBS/EDTA by repeated centrifugations at 200 g for 10 min. After washing, the cells were suspended in 100 ml RPMI 1640 with 1.5 mM l-glutamine and penicillin/streptomycin solution (PEST) (100 U/ml of penicillin and 100 μg/ml of streptomycin) (all from Life Technologies Carlsbad, CA). Thereafter, 2 ml of cell suspension was seeded onto 6-well cell culture plates (Greiner Bio-One, Frickenhausen, Germany) and allowed to adhere for 1.5 h. Nonadherent cells were removed by three washes with PBS, after which fresh RPMI 1640 supplemented with 5% fetal calf serum (FCS) and 1.5 mM l-glutamine and PEST was added. Macrophages of M0 phenotype (approximately 2 × 10^6^/well) were obtained by culturing monocytes for 7 days in RPMI 1640, 20% FCS, 1.5 mM l-glutamine, PEST, and 20 ng/ml macrophage colony-stimulating factor (M-CSF) (R&D Systems, Minneapolis, MN), with media and M-CSF renewal at day 3.

### Culture and quantification of *C. acnes*.

*C. acnes* types IA and II, previously isolated from clinical prostate cancer tissue (14), were subcultured in an anaerobic atmosphere (80% N_2_, 10% CO_2_, 10% H_2_) at 37°C on Fastidious Anaerobe Agar plates (4.6% LAB 90 Fastidious Anaerobe Agar, LAB M, Lancashire, United Kingdom) supplemented with 5% horse blood. The cultured bacteria were harvested from the plate, suspended in RPMI 1640 supplemented with 5% FCS and 1.5 mM l-glutamine, and quantified by Bürker counting.

### Stimulation of M0 macrophages with *C. acnes* types IA and II.

M0 macrophages were cultured in RPMI 1640 medium supplemented with 5% FCS and 1.5 mM l-glutamine, stimulated with 10 multiplicity of infection (MOI) of *C. acnes* type IA or type II (suspended in RPMI 1640 with 5% FCS and 1.5 mM l-glutamine), and cultured for 48 h. A total of nine experiments were performed using macrophages from three different blood donors. Each experiment included an unstimulated M0 macrophage (control), M0 macrophages stimulated with 10 MOI *C. acnes* type IA, and M0 macrophages stimulated with 10 MOI *C. acnes* type II. After 48 h of incubation the culture media were removed, filtered through a 0.22 μm filter, and stored at −20°C until being analyzed with an ELISA. The macrophages were washed with PBS before the RNA was extracted.

### RNA extraction, cDNA synthesis, and quantitative PCR.

The macrophages were lysed directly in the wells of the culture plates with RLT Plus Buffer (Qiagen, Hilden, Germany) and 2-mercaptoethanol (Sigma-Aldrich, Stockholm, Sweden) followed by the extraction procedure described in the RNeasy Plus Mini Kits (Qiagen, Hilden, Germany), eluted in 30 μl water and stored at −20°C until use. The concentration and quality of RNA in each sample was determined with spectrophotometry (Infinite M200 PRO, Tecan Trading AG, Männedorf, Switzerland), and 200 ng extracted RNA was used for cDNA synthesis using RT^2^ First Strand Kits (Qiagen, Hilden, Germany). The cDNA was diluted 1:5 and stored at −20°C. For the gene expression analysis, primers with specificity for mRNA coding for PD-L1, M-CSF, CCL2, CCL17, CCL18, and CCL22 were used (all primer pairs were validated QuantiTect primers from Qiagen Hilden, Germany) together with RT^2^ SYBR green ROX qPCR Master Mix (Qiagen, Hilden, Germany). Quantitative real-time PCR (qPCR) was performed with a StepOnePlus real-time PCR system (Applied Biosystems, ThermoFisher Scientific, USA). The qPCRs were performed in a total volume of 13 μl with 4 μl cDNA diluted 1:5 and 200 nM forward and reverse primers. Samples were analyzed in duplicate or triplicate if needed due to high variation. Each run included negative controls. *C. acnes* types IA and II cDNA were included in the qPCR with all primer pairs to confirm absence of genes with sequence similarity. The qPCR protocol consisted of initial denaturation at 95°C for 10 min, followed by a two-step cycling protocol (95°C for 15 s + 60°C for 60 s) for 40 cycles. Melting curve analyses were used to determine the identity and specificity of the PCR products. Cycle threshold was recorded for each sample, and target mRNA levels were normalized using POLR2F as reference gene (validated by Qiagen Hilden, Germany). To calculate “fold change” (i.e., the differences in gene expression level between the *C. acnes* stimulated macrophage in comparison to the unstimulated macrophage), the 2^-ΔΔCT^ method was used (45).

### ELISA.

Levels of CCL17, CCL18, and PD-L1 protein in the conditioned culture media from untreated M0 macrophage controls and *C. acnes* stimulated M0 macrophages were analyzed using pre-coated Quantikine ELISA kits (R&D Systems, Inc., Minneapolis, MN) as specified by the manufacturer. For the Quantikine ELISA human CCL17/TARC immunoassay, the samples were analyzed undiluted with a standard ranging from 31 to 2000 pg/ml. For the Quantikine ELISA human CCL18/PARC immunoassay, the samples were diluted 1:10 and analyzed with a standard ranging from 18.8 to 1200 pg/ml. For the Quantikine ELISA human/cynomolgus monkey PD-L1/B7-H1 immunoassay, the samples were analyzed undiluted at double volume using a standard ranging from 25 to 1,200 pg/ml.

### Prostate cancer tissue, *C. acnes* culture confirmation, and quantification of CD4/FOXP3+ Tregs.

In the present study, we investigated the association between the presence of *C. acnes* and infiltration of Tregs in 151 tumors obtained from 137 patients. These 137 men with prostate cancer was previously a part in a cohort of patients used by our group to investigate the presence of *C. acnes* in the prostate gland of men with and without prostate cancer (14). Among the 137 cases selected for our study, 100 cases were diagnosed with prostate cancer prior to the inclusion and 37 cases were initially categorized as controls but diagnosed with prostate cancer at the following pathological anatomical examination. The 14 additional tumors were from cases with multiple tumors. By using prostate cancer tissue from this cohort we have previously also evaluated association between infiltration of Tregs and clinical outcomes in prostate cancer (24). The Treg cell positivity were determined by CD4+FOXP3+ immunohistochemical staining. An area of 10 mm^2^ in sections of each tumor were visually inspected for presence of Tregs in both epithelia and tumor stromal area (24). By using data from these two investigations (14, 24), we now assessed the relationship between *C. acnes* and Tregs.

### Statistical methods.

Differences in mRNA expression and protein levels between *C. acnes* stimulated and unstimulated macrophages were investigated. For calculation of mRNA expression, the ΔCt values generated by the qPCR (i.e., the mRNA expression of the mRNA of interest minus the mRNA expression of the housekeeping gene POLR2F) were used. For the calculation of the change in protein level the pg/ml value generated by the ELISA was used. The mRNA and protein analyses were performed using repeated measurements ANOVA with two within subject factors, macrophage treatment and replicate. The different macrophage treatments were unstimulated (control), stimulated with *C. acnes* type IA or with *C. acnes* type II. The number of replicates from each of the three macrophage donors were three. Data were log_10_-transformed after testing for normality and homogeneity of variances using the Kolmogorov-Smirnov test and Levene’s test for equality of variances.

Chi-square tests were used to evaluate the relationship between the presence of *C. acnes* (culture positive or culture negative) and infiltration of Tregs (present or not present) in tumor epithelia and stromal area, respectively. Among the 137 cases included in our study, 73 cases were identified as *C. acnes* positive and 64 cases were identified as *C. acnes* negative. An additional 14 tumors from cases with multiple tumors were included, among theses, 10 were *C. acnes* negative and 4 were *C. acnes* positive. All analyses were conducted in versions 22 or 24 of SPSS (IBM, Armonk, NY) and version 14 of STATA (Stata Corp., College Station, TX).
