# Supplementary material for: Cutibacterium acnes Induces the Expression of Immunosuppressive Genes in Macrophages and is Associated with an Increase of Regulatory T-Cells in Prostate Cancer
Source: Microbiol Spectr. 2021 Dec 22;9(3):e01497-21. doi: 10.1128/spectrum.01497-21 (PMC8694172; doi:10.1128/spectrum.01497-21)

Raw data mean value mRNA ΔCt, macrophage donor (1-3) and repeated measures (1-3), log10 ΔCt (dCt) for CCl2, CSF1 and CCL22.

**Macrophage donor 1**	**ccl2mRNA Δct**	**ccl2 mRNA Δct+7**	**ccl2mRNA Δct+7 log10 value for SPSS**
mo k 1.1	1,458327611	8,458327611	0,9272
mo k 1.2	0,954372724	7,954372724	0,9006
mo k 1.3	0,963564237	7,963564237	0,9011
mo typI 1.1	-4,458788236	2,541211764	0,405
mo typI 1.2	-4,179164886	2,820835114	0,4503
mo typI 1.3	-3,373573303	3,626426697	0,5594
mo typII 1.1	0,497495015	7,497495015	0,8749
mo typII 1.2	0,370980581	7,370980581	0,8675
mo typII 1.3	0,442392031	7,442392031	0,8717
**Macrophage donor 2**			
mo k 2.1	-0,396636963	6,603363037	0,8197
mo k 2.2	0,194731394	7,194731394	0,857
mo k 2.3	-0,485848745	6,514151255	0,8138
mo typI 2.1	-5,509380023	1,490619977	0,1733
mo typI 2.2	-5,232415199	1,767584801	0,2473
mo typI 2.3	-6,10801665	0,89198335	-0,04
mo typII 2.1	-3,730719248	3,269280752	0,5144
mo typII 2.2	-3,173311234	3,826688766	0,5828
mo typII 2.3	-3,474483808	3,525516192	0,5472
**Macrophage donor 3**			
mo k 3.1	0,008858045	7,008858045	0,8456
mo k 3.2	-0,107248624	6,892751376	0,8383
mo k 3.3	-0,271410942	6,728589058	0,8279
mo typI 3.1	-4,23200798	2,76799202	0,4421
mo typI 3.2	-3,002209345	3,997790655	0,6018
mo typI 3.3	-3,089935621	3,910064379	0,5921
mo typII 3.1	-3,741991043	3,258008957	0,5129
mo typII 3.2	-4,059759458	2,940240542	0,4683
mo typII 3.3	-2,882748286	4,117251714	0,6146

**Macrophage donor 1**	**CSF1 mRNA Δct**	**CSF1 mRNA Δct+7**	**CSF1 mRNA Δct+7 log10 värde till SPSS**
mo k 1.1	-2,745598475	4,254402	0,6288
mo k 1.2	-3,557873408	3,442127	0,5368
mo k 1.3	-3,495406787	3,504593	0,5446
mo typI 1.1	-1,5681928	5,431807	0,7349
mo typI 1.2	-2,126134872	4,873865	0,6878
mo typI 1.3	-1,556347847	5,443652	0,7358
mo typII 1.1	-2,222839991	4,77716	0,6791
mo typII 1.2	-2,081692378	4,918308	0,6918
mo typII 1.3	-1,941713651	5,058286	0,7039
**Macrophage donor 2**			
mo k 2.1	-2,070074081	4,929926	0,6928
mo k 2.2	-2,410605748	4,589394	0,6617
mo k 2.3	-2,75329717	4,246703	0,628
mo typI 2.1	-1,975570361	5,02443	0,701
mo typI 2.2	-2,742402077	4,257598	0,6291
mo typI 2.3	-2,658816973	4,341183	0,6375
mo typII 2.1	-0,799602191	6,200398	0,7924
mo typII 2.2	-0,766606331	6,233394	0,7947
mo typII 2.3	-0,888258298	6,111742	0,7861
**Macrophage donor 3**			
mo k 3.1	-3,91168944	3,088311	0,4897
mo k 3.2	-3,401546796	3,598453	0,5561
mo k 3.3	-3,870518684	3,129481	0,4954
mo typI 3.1	-4,485412598	2,514587	0,4004
mo typI 3.2	-6,135872046	0,864128	-0,0634
mo typI 3.3	-5,606117566	1,393882	0,1442
mo typII 3.1	-3,948732376	3,051268	0,4844
mo typII 3.2	-4,149604162	2,850396	0,4548
mo typII 3.3	-3,774078051	3,225922	0,5086

Macrophage donor 1	CCL22 mRNA ∆ct	CCL22 mRNA ∆ct +7	CCL22 mRNA ∆ct+8 log10 value to SPSS
mo k 1.1	-4,016192118	3,983808	0,6002
mo k 1.2	-4,302281698	3,697718	0,5679
mo k 1.3	-4,62098821	3,379012	0,5287
mo typl 1.1	-4,648969014	3,351031	0,5251
mo typl 1.2	-4,243758519	3,756241	0,5747
mo typl 1.3	-3,886943181	4,113057	0,6114
mo typll 1.1	-4,056514104	3,943486	0,5958
mo typll 1.2	-4,302371343	3,697629	0,5679
mo typll 1.3	-4,005942663	3,994057	0,6014
**Macrophage donor 2**			
mo k 2.1	-6,06415081	1,935849	0,2868
mo k 2.2	-5,562984149	2,437016	0,3868
mo k 2.3	-5,457440058	2,54256	0,4052
mo typl 2.1	-4,544478734	3,455521	0,5385
mo typl 2.2	-4,973629634	3,02637	0,4809
mo typl 2.3	-5,374159495	2,625841	0,4192
mo typll 2.1	-3,703272502	4,296727	0,6331
mo typll 2.2	-3,864143372	4,135857	0,6165
mo typll 2.3	-3,814766566	4,185233	0,6217
**Macrophage donor 3**			
mo k 3.1	-5,795724233	2,204276	0,3432
mo k 3.2	-5,648554802	2,351445	0,3713
mo k 3.3	-5,925309181	2,074691	0,3169
mo typl 3.1	-7,605340322	0,39466	-0,4038
mo typl 3.2	-6,004000028	1,996	0,3001
mo typl 3.3	-6,442307472	1,557693	0,1924
mo typll 3.1	-6,121786753	1,878213	0,2737
mo typll 3.2	-4,995523453	3,004477	0,4777
mo typll 3.3	-5,62686348	2,373137	0,3753

mRNA ∆Ct log10 **CCL2 CSF1 and CCL22 analysis SPSS**

Repeted measures within-subject factor treatment (1-3) and replicates (1-3)

Treatment 1 (untreated macrophage)

Treatment 2 (Macrophage treated with *C. acnes* Typ IA)

Treatment 3 (Macrophage treated with *C. acnes* Type II)

CCL2 mRNA

Estimates

Measure: MEASURE_1

treatment	Mean	Std. Error	95% Confidence Interval Lower Bound	95% Confidence Interval Upper Bound
1	.859	.026	.749	.970
2	.381	.129	-.174	.936
3	.650	.111	.175	1.126

Pairwise Comparisons

Measure: MEASURE_1

(I) treatment	(J) treatment	Mean Difference (I-J)	Std. Error	Sig.[a]	95% Confidence Interval for Difference[a] Lower Bound	95% Confidence Interval for Difference[a] Upper Bound

1	2	.478	.120	.058	-.040	.996
	3	.209	.085	.134	-.157	.575
2	1	-.478	.120	.058	-.996	.040
	3	-.269	.141	.197	-.878	.339
3	1	-.209	.085	.134	-.575	.157
	2	.269	.141	.197	-.339	.878

Based on estimated marginal means

a. Adjustment for multiple comparisons: Least Significant Difference (equivalent to no adjustments).

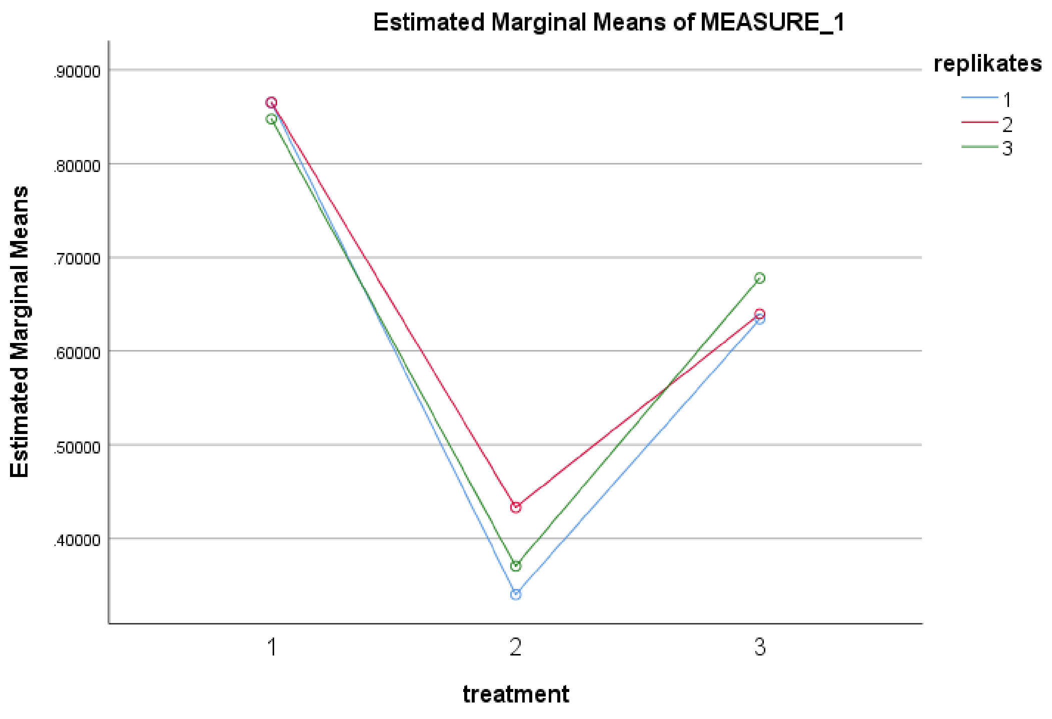

CSF1 mRNA

Estimates

Measure: MEASURE_1

treatment	Mean	Std. Error	95% Confidence Interval Lower Bound	Upper Bound
1	.582	.043	.397	.766
2	.512	.177	-.248	1.272
3	.655	.091	.264	1.046

Pairwise Comparisons

Measure: MEASURE_1

(I) treatment	(J) treatment	Mean Difference (I-J)	Std. Error	Sig.[a]	95% Confidence Interval for Difference[a]	
					Lower Bound	Upper Bound
1	2	.070	.149	.686	-.570	.709
	3	-.074	.052	.296	-.299	.152
2	1	-.070	.149	.686	-.709	.570
	3	-.143	.101	.293	-.578	.292
3	1	.074	.052	.296	-.152	.299
	2	.143	.101	.293	-.292	.578

Based on estimated marginal means

a. Adjustment for multiple comparisons: Least Significant Difference (equivalent to no adjustments).

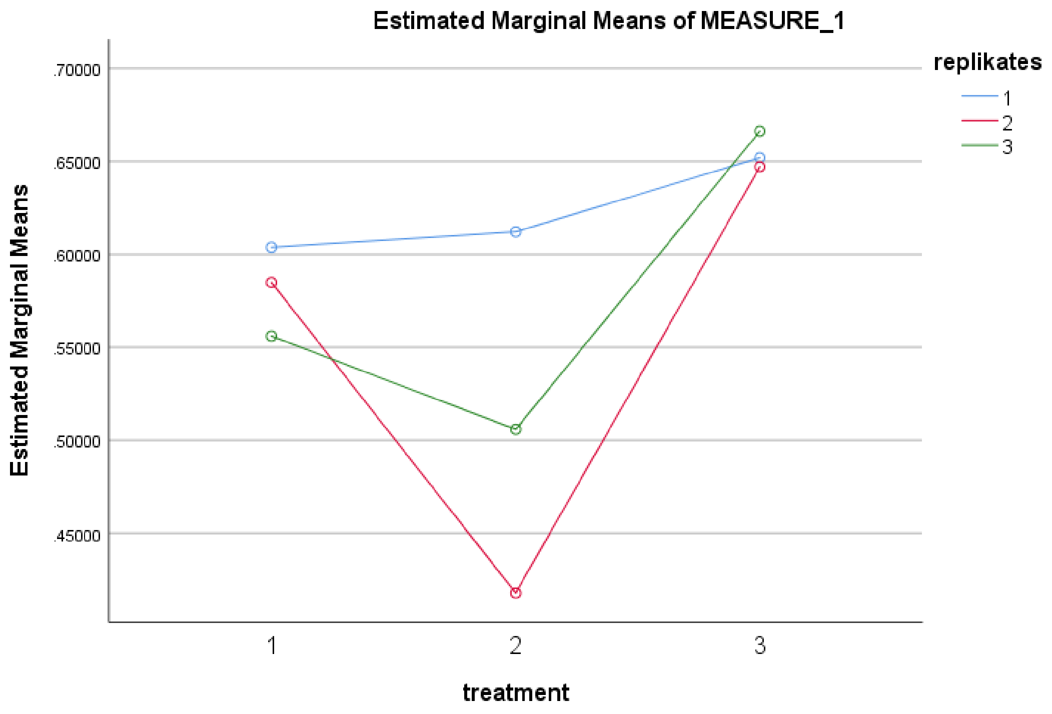

CCL22 mRNA

Estimates

Measure: MEASURE_1

treatment	Mean	Std. Error	95% Confidence Interval	
			Lower Bound	Upper Bound
1	.423	.071	.116	.730
2	.360	.167	-.360	1.079
3	.529	.078	.196	.863

Pairwise Comparisons

Measure: MEASURE_1

(I) treatment	(J) treatment	Mean Difference (I-J)	Std. Error	Sig.[a]	95% Confidence Interval for Difference[a] Lower Bound	Upper Bound
1	2	.063	.130	.675	-.496	.622
	3	-.106	.079	.311	-.446	.234
2	1	-.063	.130	.675	-.622	.496
	3	-.169	.096	.218	-.580	.242
3	1	.106	.079	.311	-.234	.446
	2	.169	.096	.218	-.242	.580

Based on estimated marginal means

a. Adjustment for multiple comparisons: Least Significant Difference (equivalent to no adjustments).

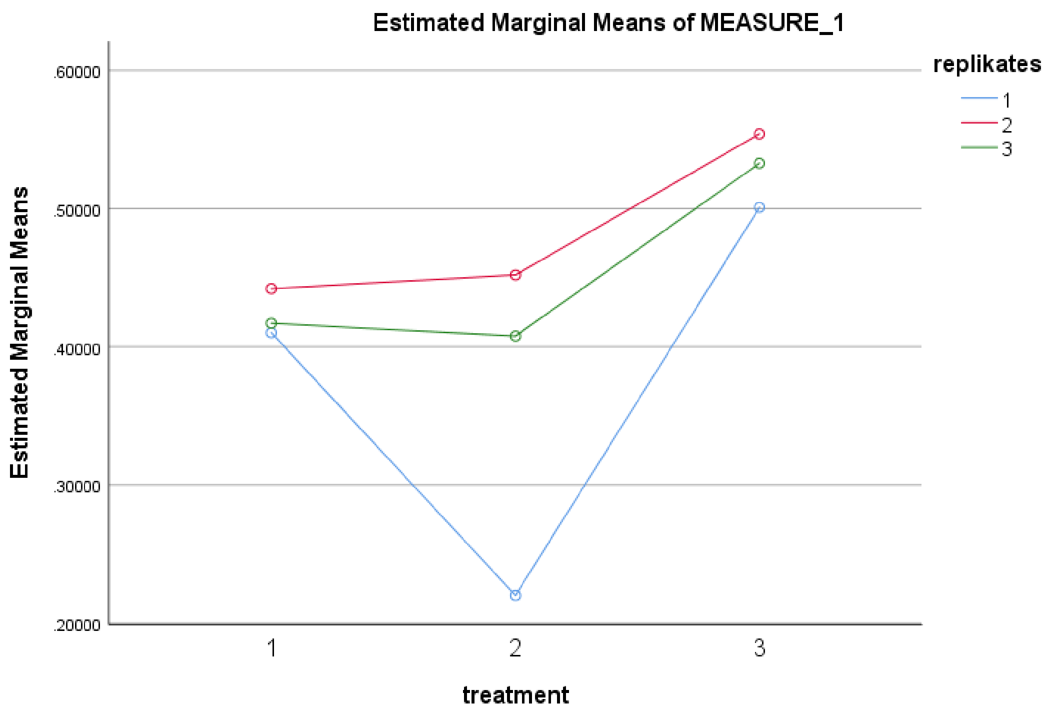

Supplement: SUPPLEMENTAL FILE 1 — Supplemental material. Download SPECTRUM01497-21_Supp_1_seq7.pdf, PDF file, 0.7 MB [file spectrum01497-21_supp_1_seq7.pdf]
